# Aneurysmal Bone Cyst of the Lateral Malleolus Treated with Intralesional Curettage and Masquelet Technique: A Case Report

**DOI:** 10.5704/MOJ.2107.025

**Published:** 2021-07

**Authors:** M Gaspar, MP Lasam, JA Carag, J Malana

**Affiliations:** Department of Orthopaedics, Cagayan Valley Medical Center, Tuguegarao City, Philippines

**Keywords:** ABC, lateral malleolus, Masquelet, case report

## Abstract

An aneurysmal bone cyst is a locally destructive lesion considered to be a pseudotumor arising from the bone. Although this benign-like lesion is generally considered rare, several approaches for treatment have been presented. We report a case involving a 15-year-old female diagnosed with aneurysmal bone cyst of the lateral malleolus. Applying the Masquelet technique enabled the treatment of the lesion without causing instability to the ankle joint and the prevention of recurrence through the application of polymethylmethacrylate. To our knowledge this is the first documented case in our region and possibly in the Philippines.

## Introduction

Aneurysmal bone cyst (ABC) is osteolytic and characterised by several sponge-like blood or serum-filled, generally non-endothelialized, spaces of various diameters. Majority arise from the proximal humerus, distal femur, proximal tibia, and the spine, with its preference being the metaphysis of long bones.

ABC is generally considered rare. It accounts for less than 6% of all bone tumours and is found to be four times rarer than osteosarcoma^1^. It is more common during the second decade of life and is a rare entity above 30 years^1,2,3^. In the Philippines, ABC only accounts for 9% of benign tumours and around 3.5% of primary bone tumours^2^.

In general, the treatment of ABC includes en bloc surgical resection, intralesional surgical procedures with or without the use of local adjuvants, minimally invasive surgical techniques, embolisation, sclerotherapy, radiotherapy, and the use of monoclonal antibodies and bisphosphonates^3,4^.

Here, we report a case of an ABC involving the lateral malleolus treated with the Masquelet technique. An intralesional debridement and curettage with the application of polymethylmethacrylate (PMMA) spacer was done. Five weeks later, the spacer was removed and replaced with autologous bone graft. After an extensive literature research, we account this case as the first to be documented in the region and possibly in the Philippines.

## Case Report

This is a case of a 15-year-old female who sought consult for right ankle pain that started three months prior, aggravated by weight-bearing activities and associated with a slowly enlarging mass on the lateral aspect of her ankle. No other symptoms nor any history of trauma was noted. Though the history may seem to be short for a benign tumour, tendency of delayed consult is inherent in our setting and must be considered.

**Fig. 1:**
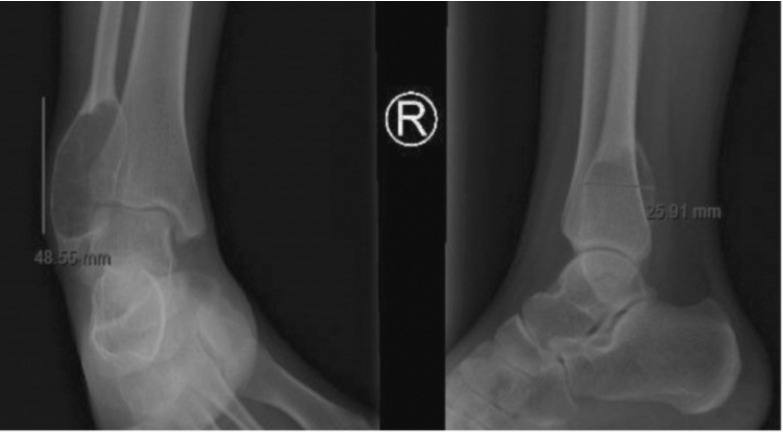
Plain radiograph of the ankle in anteroposterior and lateral views.

During examination, a mass on the right lateral malleolar area of approximately 3x4cm was noted. The mass was fixed, hard, and tender on palpation. Range of motion of the ankle was normal except for subtalar eversion at 3 degrees. Plain radiograph demonstrated a sharply defined, expansible osteolytic lesion at the lateral malleolus with thin sclerotic margins (Fig. 1). Radiographic signs were compatible with a benign tumour^2^.

**Fig. 2:**
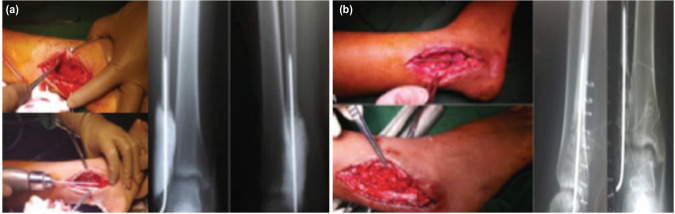
First and second stage procedure of the Masquelet technique. (a) After application of PMMA spacer. (b) Removal of the PMMA spacer and application of autologous bone.

Lateral approach to the distal fibula was done wherein the mass was removed in a piecemeal fashion (Fig. 2a) and was sent for intra-operative rush frozen section. Upon verbal confirmation of ABC, we placed a bone cement in the defect with an intramedullary pin to hold the cement spacer in place, then cauterise the lining of the cavity, and maintain the bone length and alignment. Intra-operatively, range of motion, particularly dorsiflexion and plantar flexion, was not limited. She was also placed on short leg posterior mold and non-weight-bearing crutch ambulation to prevent motion of the ankle joint. Biopsy result was consistent with ABC.

**Fig. 3:**
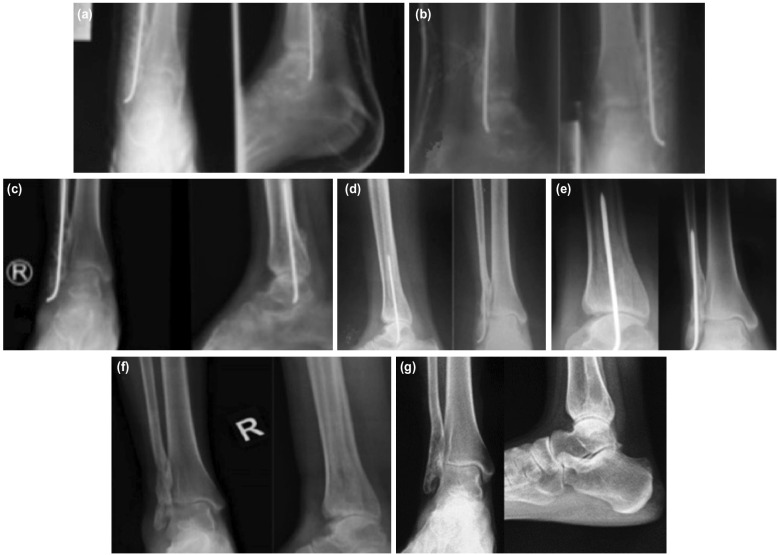
Outpatient consultations, (a) four weeks post-operative, (b) six weeks post-operative, (c) eight weeks post-operative, (d) seven months post-operative, (e) nine months post-operative, (f) post removal of implant, (g) three years post-operative.

After five weeks, the cement spacer was removed and bone graft was applied (Fig. 2b). Post-operatively, she was maintained on a non-weight-bearing crutch ambulation for six weeks (Fig. 3a). On the sixth week, the mold was removed and replaced with ankle plaster device to prevent inversion while weight-bearing (Fig. 3b). On her eighth week of follow-up, an ankle brace was applied (Fig. 3c, d).

Nine months post-operatively, evidence of consolidation was noted (Fig. 3e). We removed the pin and continued her on full weight-bearing (Fig. 3f). She was monitored regularly and no complaints at the surgical site were reported. She was later lost to follow-up and this serves as a limitation. We however managed to convince her to have a recent radiograph. No recurrence was noted (Fig. 3g).

## Discussion

The typical presentation of ABC is that of a gradually enlarging mass of a few months duration associated with pain occurring from six months to a year and involving almost all parts of the skeleton, with the humerus and femur as the most commonly affected.

The fibula is a dispensable bone; hence, wide surgical margins are theoretically more easily achievable than in other skeletal sites. However, ample resections of distal fibular lesions may be hampered by difficulties with soft tissue coverage and the possible impact on foot and ankle biomechanics^4^.

Several solutions for different pathologies have been enumerated^4^. For decades, below-knee amputation has been the main treatment for malignant tumours involving the distal fibula and tibia. Advances in surgical techniques and chemotherapy have led to the introduction of alternative, less destructive approaches. For instance, distal fibular resection without reconstruction of the lateral side of the ankle is frequently performed. In such instances, ankle stability is obtained via either soft tissue and ligament reconstruction or tibiotalar arthrodesis. In other cases, fibular resection is followed by reconstruction with allograft, autografts, pedicled vascularised epiphyseal transfers using the ipsilateral proximal fibula or a long bone graft from the iliac crest, bone transplants, or prosthetic ankle joint replacement.

Subperiosteal excision has been reported^5^ with none of the patients receiving instillation of bone marrow, autogenous bone graft, allograft, or any synthetic bone substitutes but the periosteal covering was saved, resulting to bone regeneration within three to nine months with no joint instability or recurrence.

Masquelet technique is a procedure done in two phases for the treatment of bone defects as large as 25cm. The first surgery utilises bone cement as a spacer and induces a membrane around the spacer. The second surgery entails careful incision of the induced membrane and replacement of the spacer with a cancellous bone graft. The membrane would hold the graft in place while awaiting consolidation.

In conclusion, the incidence of ABC in distal fibula is very low, treatment options are also few. Regular treatment protocol for ABC is curettage and bone grafting, however, when the lesion involves the lateral malleolus, as in this case being reported, excision is preferred followed by the application of PMMA spacer and then bone graft thereafter. Lesion at distal end of fibula is definitely a rare entity and its treatment is a complex issue since maintaining ankle stability is difficult following excision^1,4^.
